# Hand Detection in Hazardous Zones of Frozen Tuna Cutting Machines Based on an Infrared Thermopile Sensor

**DOI:** 10.3390/s26134009

**Published:** 2026-06-24

**Authors:** Zhuolin Yan, Xiongsheng Zheng, Shuo Feng, Jiahao Wang, Bin Cao

**Affiliations:** School of Marine Engineering Equipment, Zhejiang Ocean University, Zhoushan 316022, China; yanzhuolin2575@163.com (Z.Y.); fs08111@163.com (S.F.); 18638066939@163.com (J.W.); 18406560598@163.com (B.C.)

**Keywords:** thermopile sensor, hand detection, dual-domain background modeling, frozen food processing, cutting safety

## Abstract

To address the challenge of hand intrusion detection in frozen tuna cutting operations where operators wear thermal-insulating gloves, this study proposes a hand detection method based on dual-domain background modeling with absolute accuracy constraints. To tackle issues arising from low-resolution infrared arrays, such as defective pixels, random noise, and complex low-temperature backgrounds, a data preprocessing pipeline integrating defective pixel correction, exponential moving average (EMA), and median filtering is developed. A dual-domain background suppression (DDBS) strategy, combining spatial-domain and temporal-domain models with sensor absolute accuracy constraints, is employed to extract hand foregrounds under complex thermal conditions. Temperature thresholding, connected-component analysis, and hole-filling are further applied to effectively separate hands from frozen tuna. An experimental platform incorporating a Raspberry Pi 4B and an MLX90640 sensor was constructed, and a dataset comprising 1173 infrared frames was collected for validation purposes. Experimental results demonstrate that the proposed method achieves an accuracy of 94.12%, precision of 91.69%, recall of 97.55%, and F1-score of 94.53% for hand intrusion detection, with an average processing time of approximately 1.84 ms per frame. This provides a cost-effective, real-time solution for hand safety monitoring in frozen food processing operations.

## 1. Introduction

In industrial production, frozen tuna is commonly processed using band-saw cutting equipment. Due to the extremely low temperature of the material, operators are required to wear thermal-insulating gloves during cutting operations. Although these gloves effectively prevent cold-related injuries, they reduce hand dexterity and increase the risk of accidental intrusion of the operator’s hands into the hazardous area surrounding the saw blade. Therefore, the development of an efficient and reliable hand intrusion detection method is of great significance for improving operational safety during frozen tuna processing.

In recent years, with the advancement of intelligent manufacturing technologies, machine vision, intelligent sensing, and automated control techniques have been increasingly applied in the seafood processing industry. Smart sensing and remote sensing technologies have been widely employed for product quality monitoring, environmental perception, and process optimization, demonstrating the potential of low-cost sensing solutions in complex industrial environments [1]. For frozen tuna processing, extensive studies have been conducted on fish segmentation, pose estimation, intelligent classification, and precision cutting. Wang et al. proposed a deep-learning-based method for frozen tuna segmentation and pose estimation [2], while Azarmdel et al. developed artificial intelligence algorithms for fish classification and precision cutting in intelligent seafood processing systems [3]. These studies have promoted the automation and intelligent development of frozen tuna processing. However, existing research primarily focuses on product perception and processing accuracy, while comparatively limited attention has been devoted to operator safety monitoring and hazardous-zone protection. In frozen tuna cutting scenarios, achieving real-time perception and active warning of hand intrusion into hazardous areas remains a critical challenge for ensuring operational safety.

Hazardous-zone intrusion detection is a core component of active safety protection systems for cutting equipment. Conventional industrial safety measures mainly rely on mechanical guards, emergency-stop devices, and safety interlock mechanisms to reduce operational risks. However, these approaches are essentially passive protection methods and are unable to identify personnel entering hazardous areas in real time. With the development of sensing technologies, researchers have increasingly incorporated sensor-based systems into active safety protection for cutting equipment. Chiang et al. [4] proposed a computer-vision-based safety monitoring framework for industrial cutting machines, utilizing deep learning algorithms to identify unsafe operations in real time. Cavedo et al. [5] developed an electronic safety system for table saws that can detect operator approaches to hazardous zones and rapidly trigger protective responses. Teixidó et al. [6] designed a safety solution for handheld circular saws based on a controllable electromagnetic field, enabling real-time risk detection around the cutting tool. Asha et al. [7] verified the feasibility of infrared sensors for hand proximity detection and safety warning applications. These studies indicate that active safety protection systems based on sensing technologies have become an important research direction in the field of industrial cutting equipment and provide technical support for the safe automation of frozen food processing.

Thermopile array sensors can acquire temperature distribution information of target surfaces in a non-contact manner. Compared with conventional infrared thermal cameras, these sensors offer advantages such as low cost, compact size, low power consumption, and broad applicability. With the development of MEMS technology, thermopile array sensors have been widely applied in fall detection [8,9,10,11], sitting posture recognition [12], human target perception [13,14], and traffic flow monitoring [15], demonstrating considerable application potential.

Human target detection is a fundamental prerequisite for risk identification, behavior analysis, and active warning in infrared-sensing-based safety monitoring systems. Extensive research has been conducted on infrared-based human detection technologies. Early approaches mainly employed passive infrared (PIR) sensors or low-resolution thermopile arrays to determine human presence and occupancy [13,16]. These methods provide advantages such as low cost, low power consumption, and strong privacy protection; however, they generally offer only coarse-grained human information and are incapable of accurate localization and target segmentation. With the advancement of infrared imaging devices, conventional machine vision techniques have been introduced into infrared human detection. By combining grayscale, gradient, and texture features with machine-learning classifiers, reliable human recognition has been achieved [17]. In addition, background differencing and adaptive background subtraction methods have been widely adopted to suppress environmental interference and improve detection performance [18,19]. More recently, the rapid development of deep learning has further advanced infrared human detection technologies. Numerous studies based on lightweight YOLO networks have achieved real-time human detection under nighttime and low-visibility conditions through multi-scale feature fusion and attention mechanisms [20,21,22]. Furthermore, infrared human detection has been extended to applications such as target tracking and abnormal behavior recognition, providing essential support for intelligent security systems and general industrial safety monitoring [23,24,25].

Despite the substantial progress achieved by existing studies, most of them focus on pedestrian monitoring, intelligent buildings, transportation systems, and general industrial environments, which differ significantly from frozen tuna cutting scenarios. First, thermal-insulating gloves substantially weaken the thermal radiation characteristics of human hands, reducing the thermal contrast between the target and the background. Second, the large low-temperature regions formed on the surface of frozen tuna can easily be confused with hand targets, thereby increasing the difficulty of foreground extraction. Moreover, industrial applications impose strict requirements on system cost and real-time performance, requiring detection algorithms to operate reliably on resource-constrained embedded platforms. Meanwhile, the large temperature difference between frozen materials and ambient air in frozen food processing environments often causes visible-light vision systems to suffer from lens condensation, surface reflections, and illumination variations, thereby reducing detection stability. Consequently, existing human detection methods developed for conventional industrial scenarios cannot be directly transferred to frozen food cutting environments.

To address these challenges, this study proposes a hand detection method for hazardous-zone intrusion monitoring in frozen tuna cutting equipment based on the MLX90640 thermopile array sensor. First, a preprocessing pipeline consisting of defective-pixel correction and hybrid filtering is applied to improve the quality of the raw infrared data. Subsequently, a Dual-Domain Background Suppression (DDBS) strategy integrating spatial-domain modeling, temporal-domain modeling, and sensor absolute accuracy constraints is proposed to extract hand foregrounds from complex thermal backgrounds. Then, temperature threshold constraints and connected-component analysis are employed to distinguish gloved hands from frozen tuna targets. Finally, the proposed algorithm is deployed on a Raspberry Pi 4B platform and evaluated using a dataset collected from a simulated frozen tuna cutting environment.

The main contributions of this study are summarized as follows:

(1) A low-cost hand detection scheme based on a thermopile array sensor is proposed to address the challenges of weak hand thermal signatures and strong low-temperature background interference in frozen tuna cutting scenarios.
(2) A dual-domain background suppression (DDBS) strategy combining spatial modeling, temporal consistency analysis, and sensor absolute accuracy constraints is developed to improve foreground extraction under complex thermal backgrounds.
(3) Real-time deployment is achieved on a resource-constrained embedded platform, providing a practical solution for the engineering implementation of active safety protection systems in frozen food processing equipment.

## 2. Materials and Methods

### 2.1. Experimental Detection Platform

**Figure 1:**
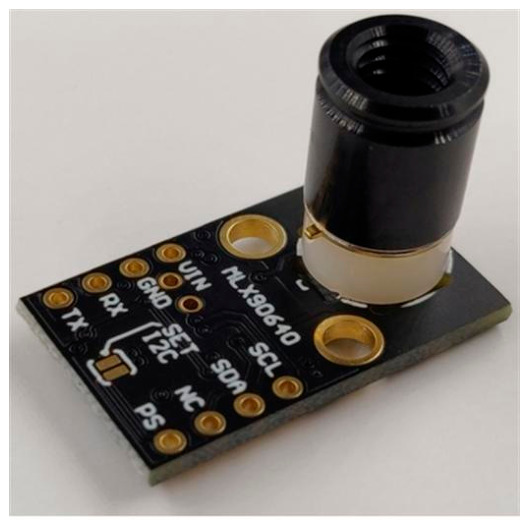
MLX90640-BAB sensor.

In this paper, the MLX90640-BAB infrared thermopile sensor (Melexis Corporation, Ieper, Belgium) was used to construct the detection system. The physical appearance and structure of the sensor are shown in Figure 1, and its main technical specifications are listed in Table 1.

**Figure 2:**
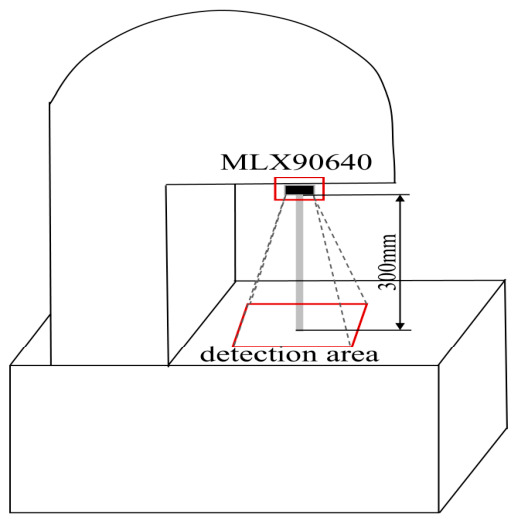
Sensor installation position.

The system employed a Raspberry Pi 4B as the core controller. The MLX90640-BAB sensor was fixed approximately 30 cm above the cutting tool of the machine to enable real-time temperature monitoring of the operational area surrounding the cutting tool. The installation method of the sensor is illustrated in Figure 2.

**Figure 3:**
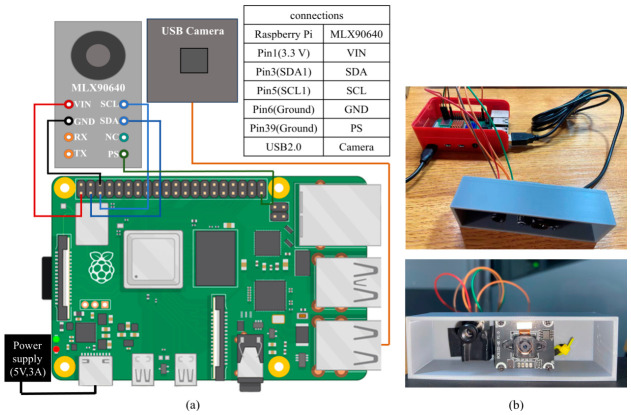
Hardware connection. (**a**) Connection schematic diagram; (**b**) Physical setup.

The hardware connection between the Raspberry Pi 4B (Raspberry Pi Trading Ltd., Cambridge, UK) and the thermopile sensor is shown in Figure 3, together forming a low-cost non-contact hand safety detection platform suitable for frozen tuna cutting scenarios.

### 2.2. Data Processing Workflow

**Figure 4:**
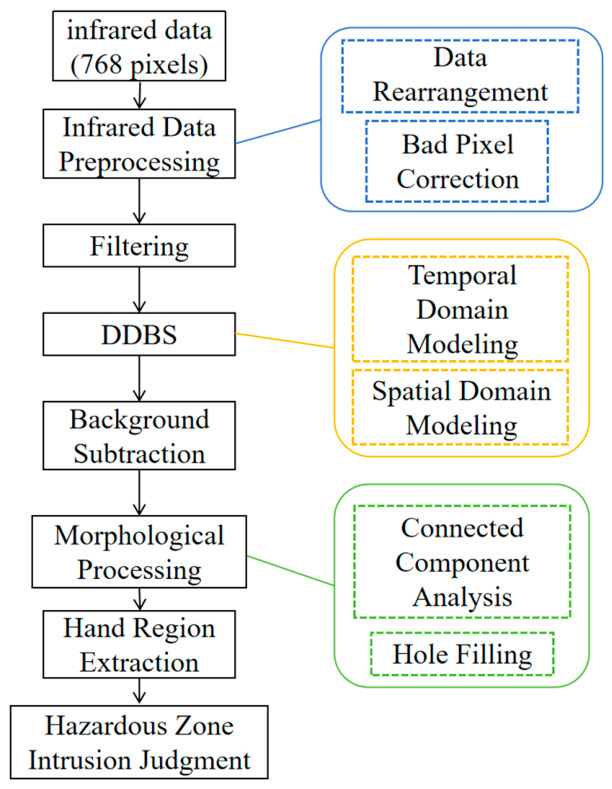
Overall workflow of the proposed hand intrusion detection method for hazardous-zone monitoring in frozen tuna cutting equipment.

The overall workflow of the proposed hazardous-zone hand intrusion detection method for frozen tuna cutting equipment is illustrated in Figure 4. The framework consists of six major stages: infrared data acquisition, data preprocessing, filtering and denoising, dual-domain background suppression, hand region extraction, and hazardous-zone intrusion detection.

The system first acquires a 24 × 32 temperature matrix (768 pixels) from the MLX90640 thermopile array sensor. As illustrated in Figure 4, the raw infrared data undergo preprocessing, including defective-pixel correction and data rearrangement, to eliminate invalid measurements and ensure consistency between the temperature matrix and the actual scene. The preprocessed data are then filtered using median and exponential moving average (EMA) filters to suppress sensor noise and environmental interference while preserving target thermal characteristics.

Subsequently, the filtered temperature data are processed using the proposed Dual-Domain Background Suppression (DDBS) framework. The spatial domain employs a multi-frame statistical background model, whereas the temporal domain analyzes inter-frame temperature variations to identify stable background regions. By jointly exploiting spatial and temporal information, the DDBS framework effectively suppresses thermal background interference and extracts foreground candidate regions.

The extracted foreground candidates are further refined through temperature thresholding, connected-component analysis, and hole-filling operations to remove cold-object interference and isolated noise, thereby enabling accurate hand-region extraction. Finally, a hazardous zone is predefined according to the geometry of the cutting equipment. Hazardous-zone intrusion is determined by evaluating the spatial overlap between the detected hand region and the predefined hazardous area. When an overlap occurs, the system identifies a hazardous intrusion event and issues a safety warning.

### 2.3. Infrared Data Preprocessing

**Figure 5:**
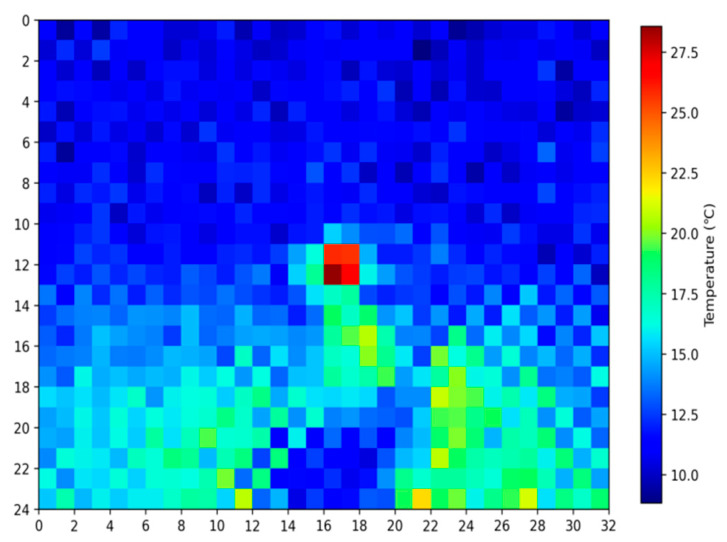
Output of the sensor.

The temperature measurement range of the thermopile sensor fully covers the thermal signatures of frozen tuna, the gloved human hand, and the ambient environment. The sensor provides a 24 × 32 pixel array. As shown in Figure 5, which displays the operating plane of the frozen tuna cutting machine, a pair of hands wearing cold-proof gloves is placed on the plane. In the thermal image, red indicates regions of higher temperature, whereas blue denotes regions of lower temperature. To enhance visualization, nearest-neighbor interpolation is applied to the infrared array during imaging, scaling it by a factor of 20 to a resolution of 480 × 640.

**Figure 6:**
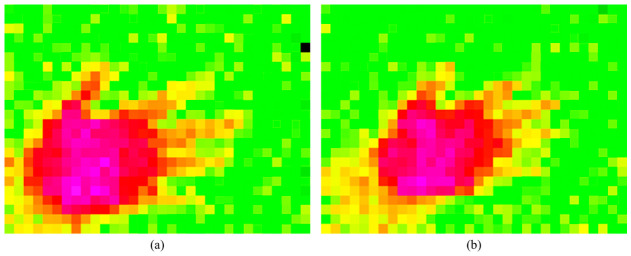
Defective pixel removal. (**a**) Before defective pixel removal, where defective pixels affect the thermal image quality; (**b**) After defective pixel removal. The heatmap represents temperature distribution, where warmer colors indicate higher temperatures and cooler colors indicate lower temperatures.

**Figure 7:**
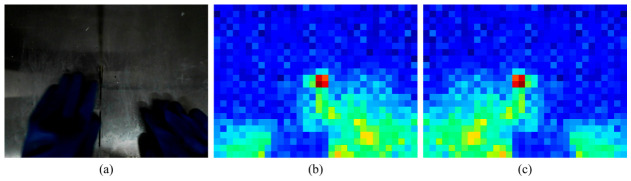
Comparison of thermal images before and after data remapping. (**a**) Visible-light image of the scene; (**b**) Thermal array image before data remapping (mirrored relative to the actual scene); (**c**) Thermal array image after data remapping (aligned with the visible-light perspective). The heatmap represents temperature distribution, where warmer colors indicate higher temperatures and cooler colors indicate lower temperatures.

The MLX90640 infrared array sensor inherently possesses a certain number of fixed invalid pixels (defective pixels) ex-factory, and its original pixel arrangement exhibits a mirrored distribution compared with conventional visible-light images. These characteristics directly affect the accuracy of subsequent temperature field analysis and target localization. Therefore, before performing subsequent algorithmic processing, the raw infrared data were first preprocessed. On one hand, the manufacturer’s official method was adopted to remove and compensate for inherent defective pixels; a comparison of the thermal images before and after defective pixel correction is shown in Figure 6. On the other hand, pixel index remapping was performed to align the spatial distribution of the infrared temperature field with the visible-light imaging perspective; the comparison before and after correction is also illustrated in Figure 7.

### 2.4. Filtering and Denoising

In data acquisition based on the MLX90640 infrared array sensor, random noise and local fluctuations are inevitably present in the infrared temperature matrix due to factors such as sensor thermal noise, quantization errors, and communication jitter. Failure to suppress these noises may lead to unstable background modeling and blurred edges of foreground targets, thereby compromising the accuracy of subsequent hand target recognition. Therefore, it is necessary to perform filtering and denoising on the infrared image after defective pixel removal.

Median filtering is a typical nonlinear spatial domain filtering method. By sorting the pixel values within the neighborhood of a given pixel and replacing the original value of the center pixel with the median value, it effectively suppresses abnormal fluctuations and isolated noise points. The computational procedure of median filtering is as follows:

1. Arrange all pixel values within a k × k window (where k is an odd number) in ascending order;
2. Select the middle pixel value as the median value.

Mean filtering is a typical linear spatial domain filtering method. Its fundamental principle is to replace the current pixel value with the average temperature of the pixels within its neighborhood, thereby achieving smoothing of random noise in the image. In the mean filtering process, a k × k neighborhood window (where k is an odd number) is selected, the arithmetic mean of all pixel values within this window is computed, and the resulting value is taken as the new filtered value for that pixel. The mathematical expression can be formulated as follows:

(1) $${\bar{T}}_{t}(i,j)=\frac{1}{k^{2}}\sum_{u=-k/2}^{k/2}\sum_{v=-k/2}^{k/2}T_{t}\left(i+u,j+v\right)$$

**Figure 8:**
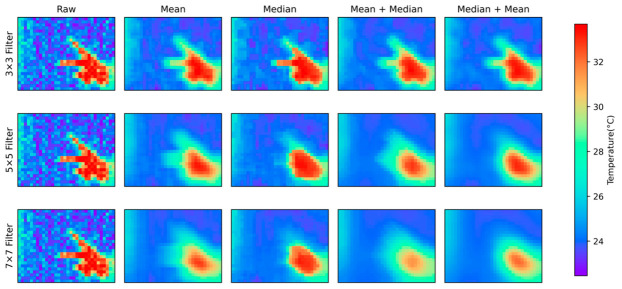
Comparative analysis of filtering algorithms.

Figure 8 compares the performance of median filtering, mean filtering, and their sequential combinations. The first column presents the unfiltered pseudo-color image as a reference. The second and third columns show the results of applying mean filtering and median filtering independently, respectively. The fourth and fifth columns present the results of applying mean filtering followed by median filtering and median filtering followed by mean filtering, respectively. Three different window sizes—3 × 3, 5 × 5, and 7 × 7—were evaluated for each filtering configuration.

It can be intuitively observed from Figure 8 that the use of 5 × 5 and 7 × 7 filtering windows results in a substantial loss of detailed information. Moreover, the combination of median and mean filtering does not yield significantly better performance than either filter applied individually. Consequently, for further algorithm comparisons, only single median filtering or single mean filtering with a 3 × 3 window is considered in the spatial domain.

**Figure 9:**
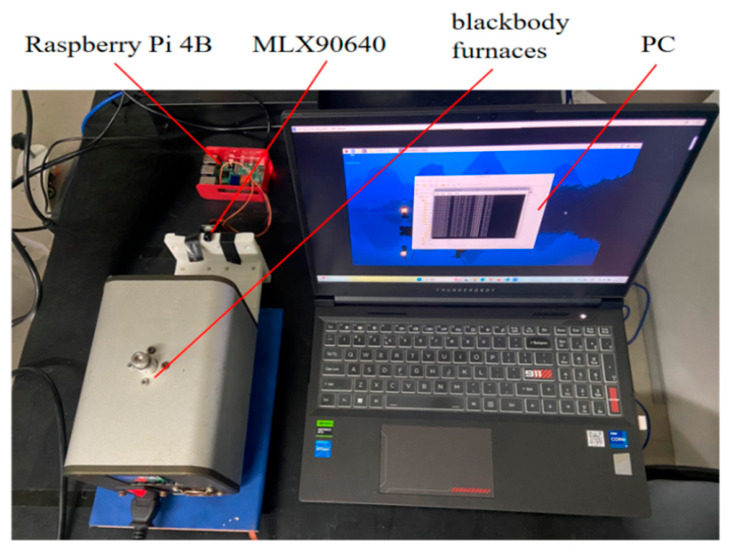
Blackbody temperature acquisition experimental scenario.

To quantitatively evaluate the denoising performance of different filtering algorithms, the per-pixel normalized root mean square error (NRMSE) was calculated following the infrared sensor noise performance evaluation method provided in the MLX90640 datasheet. In this study, the NRMSE is not intended to characterize the intrinsic performance of the sensor, but rather to compare the effectiveness of different filtering schemes in suppressing noise while preserving temperature information relevant to target detection. Figure 9 illustrates the experimental setup, in which the MLX90640 sensor was positioned at a fixed location facing a blackbody source at a controlled temperature.

**Figure 10:**
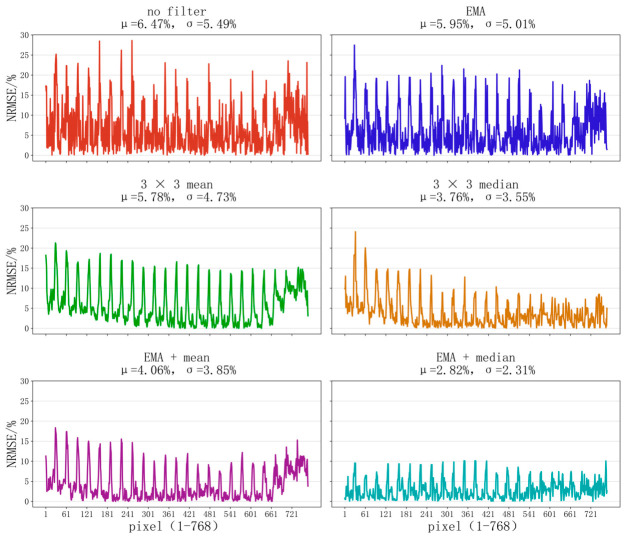
NRMSE comparison of multiple filtering algorithms.

Specifically, the blackbody temperature was set to 15 °C, and the sensor was placed at a distance of 9 cm from the blackbody. Following defective pixel correction, temperature data were collected under six conditions: (1) no filtering; (2) temporal 2-frame exponential moving average (EMA) filtering; (3) spatial 3 × 3 mean filtering; (4) spatial 3 × 3 median filtering; (5) temporal EMA combined with spatial 3 × 3 mean filtering; (6) temporal EMA combined with spatial 3 × 3 median filtering. Pixel-wise NRMSE calculations and noise analysis were then performed. The resulting NRMSE comparisons are presented in Figure 10, and the quantitative analysis results are summarized in Table 2.

The calculation formula of Exponential Moving Average (EMA) is as follows:

(2) $${CURRENT}_{EMA}={CURRENT}_{value}\times \alpha +{PREVIOUS}_{EMA}\times (1-\alpha )$$

${CURRENT}_{EMA}$ is the EMA-filtered value of a pixel in the current frame, ${CURRENT}_{value}$ is the original pixel value in the current frame, ${PREVIOUS}_{EMA}$ is the EMA-filtered value of the same pixel in the previous frame, and α (0 < α < 1) is the smoothing factor.

The smoothing factor α determines the trade-off between noise suppression capability and dynamic response speed. A smaller α provides stronger smoothing performance but may introduce response lag, whereas a larger α enables more sensitive detection of abrupt temperature changes but is more susceptible to random noise. In frozen tuna processing scenarios, hand movements are relatively slow, and the primary objective of filtering is to suppress random fluctuations in thermal imaging data. Therefore, a value of α = 0.3 was selected in this study as a compromise between filtering stability and target dynamic response performance.

The pixel-wise normalized root mean square error (NRMSE) is calculated using the following formula:

(3) $${\mathrm{NRMSE}}_{i}=\frac{\sqrt{{(T_{i}\;-\;K)}^{2}}}{K}\times 100\%$$

where ${\mathrm{NRMSE}}_{i}$ denotes the normalized root mean square error of the i-th pixel, $T_{i}$ is the measured temperature value of the i-th pixel, and K is the set blackbody temperature serving as the reference value.

The mean NRMSE is calculated using the following formula:

(4) $$\mu_{\mathrm{NRMSE}}\;=\;\frac{\sum_{i\;=\;1}^{768}{\mathrm{NRMSE}}_{i}}{768}$$

Here, $\mu_{\mathrm{NRMSE}}$ represents the average NRMSE over the 768 pixels of the sensor array. This metric measures the overall error level across all pixels and reflects the global accuracy of the filtering algorithm. A smaller $\mu_{\mathrm{NRMSE}}$ indicates higher overall algorithmic accuracy.

The noise standard deviation is calculated using the following formula:

(5) $$\sigma_{\mathrm{NRMSE}}\;=\;\sqrt{\frac{\sum_{i\;=\;1}^{768}{\left({\mathrm{NRMSE}}_{i}\;-\;\mu_{\mathrm{NRMSE}}\right)}^{2}}{768}}$$

$\sigma_{\mathrm{NRMSE}}$ represents the standard deviation of the NRMSE over the 768 pixels of the sensor array. This metric measures the fluctuation degree of the NRMSE among pixels and reflects the denoising performance of the algorithm. A smaller $\sigma_{\mathrm{NRMSE}}$ indicates better denoising effectiveness of the algorithm.

Based on the comparison of the mean NRMSE ($\mu_{\mathrm{NRMSE}}$) obtained from the quantitative analysis of the filtering algorithms, it can be observed that the data processed by the EMA combined with the spatial median filtering algorithm exhibits the overall temperature closest to the set blackbody value, indicating that this algorithm achieves the highest accuracy in restoring the true temperature value. From the comparison of the noise standard deviation, it can be seen that under this algorithm, the temperature distribution of the infrared array sensor is the smoothest, demonstrating the best suppression effect on random noise.

### 2.5. Absolute Accuracy Calculation

In the actual infrared data acquisition process, the measurement results of the sensor are inevitably affected by electronic noise, environmental interference, and the sensor’s inherent measurement errors. Even under completely static background conditions, pixel temperature values may still exhibit a certain degree of random fluctuation between consecutive frames. If the absolute consistency of pixel values is directly used as the criterion for background determination, such minor variations caused by noise can be easily misjudged as real change regions, thereby reducing the stability of background screening. Therefore, it is necessary to incorporate the measurement accuracy of the sensor and reasonably establish a temperature variation threshold to achieve a precise distinction between background and foreground.

To obtain a reliable temperature error range, experimental calibration of the infrared array sensor was conducted under constant-temperature blackbody conditions. The experimental setup was the same as shown in Figure 8. The blackbody temperature was set to 15 °C, and a total of 402 frames of infrared temperature data, which had undergone defective pixel correction, outlier removal, and filtering, were continuously acquired. Subsequently, the frame’s absolute accuracy was calculated using the following formula:

(6) $$T\_\mathrm{frame}\;=\;\frac{\sum_{m\;=\;1}^{768}T(m)}{768}$$

(7) frame_acc=|T_frame−Target_temp|

(8) non_uniformity=max(|T(m)−T_frame|)

(9) abs_acc=frame_acc+non_uniformity

where T_frame is the frame average value (the average of the 768 pixels in the current frame), frame_acc is the frame accuracy (the difference between the average value of the current frame and the actual temperature), non_uniformity is the non-uniformity (the maximum absolute deviation between the temperature values of the 768 pixels and the frame average), and the absolute accuracy is defined as the sum of the frame accuracy and the non-uniformity.

After removing the maximum and minimum frames from a total of 402 infrared frames, the temperature deviations of all remaining 400 frames’ pixels were statistically analyzed. The average absolute deviation of the sensor pixel temperatures was determined to be approximately 2.28 °C. Based on this analysis, the pixel temperature variation threshold for background determination was set to 2.3 °C. This threshold not only accounts for the intrinsic measurement fluctuations of the sensor but also effectively suppresses false detections caused by noise, while fully preserving foreground targets exhibiting significant temperature variations. Therefore, the absolute accuracy analysis provides a reliable parameter basis for background modeling, making the infrared data preprocessing and foreground extraction processes more robust and precise.

### 2.6. Dual-Domain Background Modeling

To address the infrared detection requirements in frozen tuna cutting scenarios, a dual-domain background modeling method (DDBS) was employed. This method integrates spatial-domain and temporal-domain modeling to robustly extract foreground targets under complex thermal conditions.

#### 2.6.1. Spatial-Domain Modeling

To meet the infrared detection requirements of frozen tuna cutting operations, a multi-frame statistical method was adopted to construct the background model, enabling stable and reliable characterization of the background temperature distribution. The core idea of this method is to collect several frames of infrared data without target interference during the system initialization stage and perform statistical processing to obtain the background temperature value of each pixel under stable environmental conditions. Compared with conventional single-frame background modeling methods, multi-frame statistical modeling can effectively suppress the influence of random noise, significantly improving the stability and reliability of the background model and providing a solid foundation for subsequent foreground target detection.

The frame rate of the MLX90640 infrared array sensor was set to 16 Hz, which provides a balance between infrared imaging stability and data acquisition speed. During system initialization, eight consecutive frames of environmental background data without human presence or other significant heat sources within the sensor field of view were automatically collected. At a frame rate of 16 Hz, acquiring eight background frames requires only 0.5 s. Such a short acquisition window effectively avoids the effects of gradual environmental temperature drift and low-frequency airflow disturbances, ensuring that the sampling process remains under steady-state environmental conditions. Each frame contains 768 pixels, which can be represented as a two-dimensional temperature matrix $T_{t}(i,j)$, where i and j denote the row and column coordinates of the pixel in the array, respectively, and t is the frame index. Considering that simple multi-frame averaging is susceptible to individual outliers, the extreme-value-removed averaging method was adopted to compute the background model, thereby improving the accuracy of the background temperature values. For each pixel position (i,j), the filtered temperature data from the eight frames were first collected and sorted in ascending order. To mitigate the influence of random noise and transient fluctuations on the modeling results, the maximum and minimum values after sorting were excluded from the averaging calculation, and only the middle six data points were averaged. The resulting value was taken as the background temperature value for that pixel. Through the above operations, a background temperature matrix capable of describing the stable temperature distribution within the field of view of the infrared array sensor under the current environmental conditions was obtained. This matrix serves as the Reference Background for subsequent human detection algorithms. When the difference between the temperature of a pixel in the current frame and the corresponding pixel temperature in the reference background frame is less than 2.3 °C, the temperature variation at that pixel is considered to be a random fluctuation within the sensor measurement error range and is not regarded as a real change; the pixel region is classified as background. Conversely, when the temperature difference exceeds this threshold, the region is considered to exhibit a genuine temperature change.

#### 2.6.2. Temporal-Domain Modeling

In infrared imaging systems, moving targets typically exhibit noticeable temperature variations across consecutive frames, whereas stationary background regions remain relatively stable. Therefore, temporal information can be exploited to distinguish dynamic targets from static background interference.

**Figure 11:**
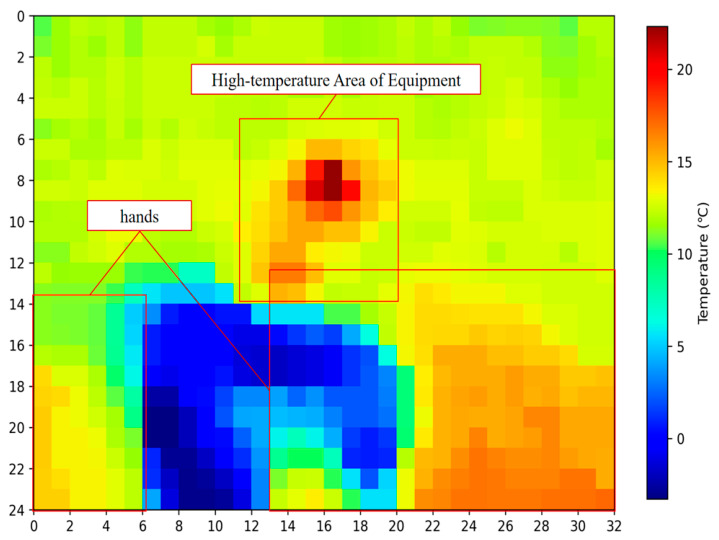
Temperature distribution of equipment hot regions and hand targets.

In the frozen tuna cutting environment, the infrared sensor captures not only the operator’s hands and frozen tuna but also several high-temperature regions on the cutting equipment. As shown in Figure 11, the temperatures of some equipment surfaces are comparable to, or even higher than, those of the operator’s hands. Consequently, temperature intensity alone is insufficient for reliable hand detection. However, unlike the operator’s hands, these equipment heat sources remain spatially stationary during operation. This observation indicates that temporal consistency can provide an effective cue for separating hand targets from static thermal interference.

Based on this analysis, a temporal-domain background model is constructed, and a time-consistency-based background determination strategy is introduced during foreground extraction. This strategy compares pixel temperature variations between consecutive frames to identify areas that remain stable over short intervals as background, thereby suppressing interference from static heat sources and focusing detection on regions with pronounced dynamic changes. Specifically, for each pixel in a continuous infrared image sequence, the temperature difference between the current frame and the corresponding pixel in the previous frame is calculated. Pixels whose temperature change is below the defined threshold are considered within the sensor’s measurement error range, judged as unchanged, and classified as temporal-domain background. Conversely, pixels exceeding the threshold are deemed to exhibit actual temperature changes and are retained for subsequent foreground target analysis. This temporal-domain background filtering strategy effectively suppresses interference from fixed high-temperature equipment regions while highlighting foreground targets such as operator hands that exhibit motion characteristics.

Regions that are not classified as background by either the spatial-domain or temporal-domain background models are retained as Prospective Candidate Regions. These regions exhibit significant temperature deviations from the reference background and observable temporal variations across consecutive frames, making them more likely to correspond to hand targets and therefore serving as the primary focus of subsequent detection procedures.

### 2.7. Hand Detection Methods

Background removal is a common data processing technique aimed at eliminating irrelevant background elements from images, documents, or other data sources to highlight foreground information, improve data quality, or optimize algorithm performance. The core objective of infrared image background removal is to separate the target region.

Considering the actual temperature distribution characteristics of the frozen tuna cutting scenario, the temperature of the frozen tuna being cut is typically at a relatively low level, while the temperature of the operator’s hand is significantly higher than that of the frozen tuna, exhibiting a distinct temperature difference between the two. Leveraging this characteristic, after obtaining the prospective candidate regions, a temperature lower-limit constraint is further introduced to perform secondary screening of the foreground regions to eliminate irrelevant interference. Specifically, for each pixel in the prospective candidate region, if its temperature is below 5 °C, it is determined to be a low-temperature region and is ignored; only regions with temperatures above 5 °C are retained as the final targets of interest. This screening step effectively eliminates interference caused by low-temperature background regions and the frozen tuna body itself, focusing recognition on high-temperature targets such as the human hand, thereby further enhancing the accuracy and specificity of target detection.

**Figure 12:**
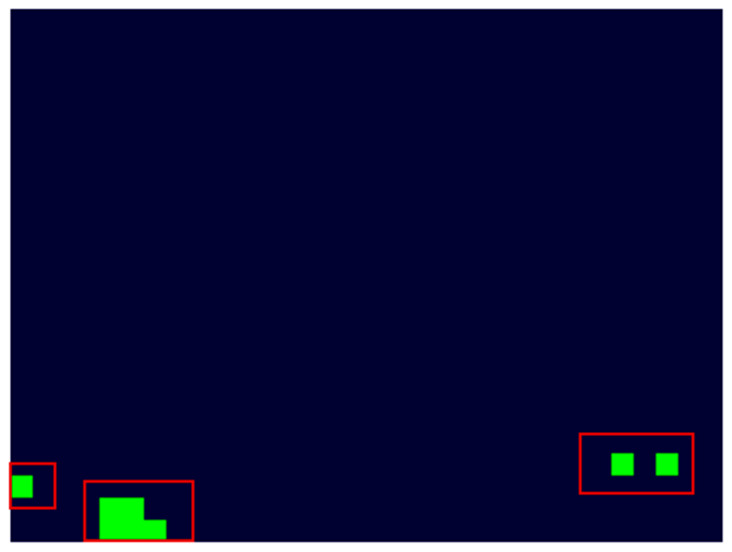
Foreground region noise, where the green region inside the red bounding box.

To further improve the reliability and integrity of the foreground regions, connected-component filtering and hole-filling processing are introduced to classify and optimize the foreground regions. First, connected-component analysis is performed on the foreground image obtained after temperature threshold segmentation. By labeling the spatial connectivity relationships among foreground pixels, the image is partitioned into several independent connected regions. Considering that the human hand typically appears as a continuous region with a certain spatial scale in infrared images, whereas pseudo-foreground regions caused by noise or local temperature anomalies are mostly scattered and small in area, as shown in Figure 12.

Foreground regions are screened based on the area characteristics of the connected components. Regions with fewer than six connected pixels are determined to be non-target regions and are eliminated, while connected regions with areas consistent with the hand scale characteristics are retained as effective foreground candidate regions. Through this process, isolated noise regions generated by threshold segmentation are effectively suppressed, and the spatial consistency of the foreground regions is improved.

**Figure 13:**
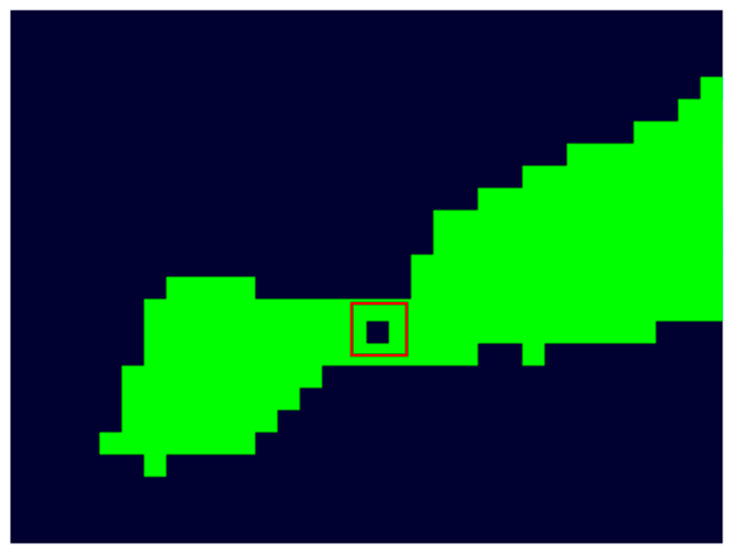
Holes in foreground regions, where the black region inside the red bounding box.

On the basis of connected-component filtering, hole-filling processing is further performed to address the local hole phenomenon that appears inside foreground regions, as shown in Figure 13. Holes are typically caused by local temperatures of the human body being slightly lower than the threshold, resulting in discontinuities within the target region. In this paper, by detecting background pixels inside the connected regions and analyzing their neighborhood connectivity relationships, background pixels surrounded by foreground regions are filled, thereby restoring the overall continuity of the target region. This processing improves the completeness and morphological consistency of the foreground regions without significantly introducing additional false detections.

**Figure 14:**
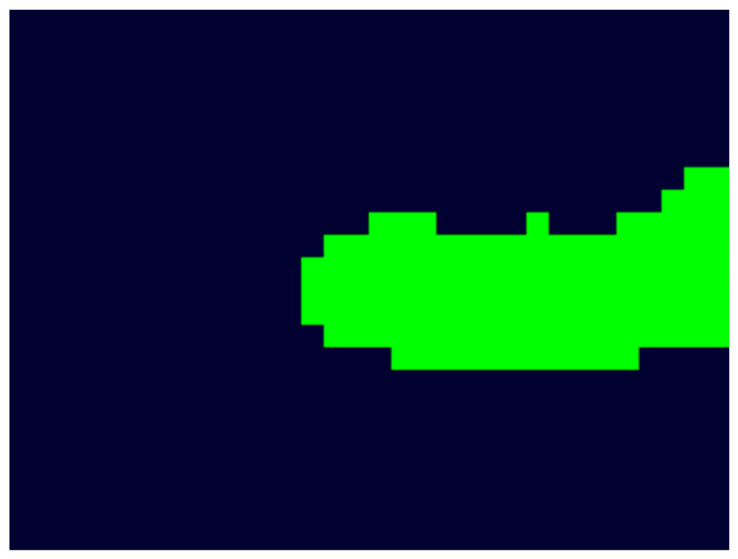
Foreground image after connected domain filtering and hole filling (the green region represents the hand, and the black region represents the background).

After the three steps of background and low-temperature region removal, connected-component filtering, and hole filling, the foreground regions in the infrared image are significantly improved in terms of spatial structure and semantic integrity, as shown in Figure 14.

### 2.8. Hazardous Zone Intrusion Detection

After extracting the hand regions from the infrared array images, the proposed method focuses solely on hand detection and hazardous intrusion judgment, without performing fine-grained multi-object classification of the foreground. With reference to the physical structure of the cutting machine, the hazardous zone is calibrated within the sensor’s imaging field. A hazardous intrusion event is identified by evaluating the spatial relationship between the detected hand region and a predefined hazardous zone associated with the cutting tool.

In practical industrial environments, hazardous zones are closely related to equipment geometry and cutting-tool locations. Therefore, a manual calibration strategy is adopted to define the hazardous zone within the infrared image coordinate system. Specifically, a fixed rectangular region is designated according to the position of the cutting tool within the sensor’s field of view. The calibrated hazardous zone is represented as $D=\{(x,y)\;|{\;x}_{d}^{\min}\leq x\leq x_{d}^{\max},y_{d}^{\min}\leq y\leq y_{d}^{\max}\}$, where $x_{d}^{\min}$, $x_{d}^{\max}$, $y_{d}^{\min}$, and $y_{d}^{\max}$ denote the boundaries of the hazardous area. The calibration is performed prior to system deployment and remains unchanged during operation, ensuring a clear correspondence between the monitored region and the actual cutting-tool location.

**Figure 15:**
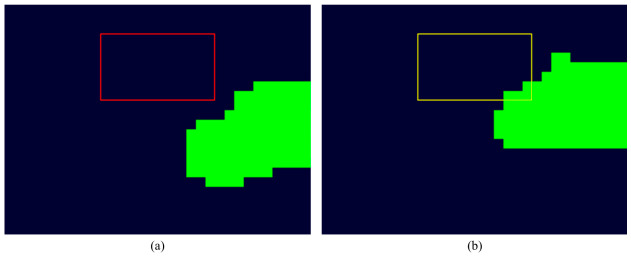
Comparative analysis of hazard zone intrusion detection, where the green region represents the hand and the black region represents the background. (**a**) Hand outside the predefined hazardous zone, the hazardous zone boundary is shown in red; (**b**) Hand inside the predefined hazardous zone, the hazardous zone boundary is shown in yellow.

Figure 15 illustrates the intrusion evaluation process. The rectangle represents the calibrated hazardous zone, while the green region corresponds to the extracted hand target. When the detected hand region remains outside the hazardous zone, the situation is classified as safe. Conversely, if any portion of the hand region overlaps with the hazardous zone, the system identifies the event as a hazardous intrusion. This overlap-based strategy enables direct and computationally efficient assessment of potential safety risks while maintaining clear engineering interpretability.

### 2.9. Performance Evaluation Metrics

To evaluate the performance of the proposed detection method, four widely used metrics, namely Accuracy, Precision, Recall, and F1-Score, were adopted in this study. The corresponding formulas are given as follows:

(10) $$Acc=\frac{\mathrm{TP}\;+\;\mathrm{TN}}{\mathrm{TP}\;+\;\mathrm{TN}\;+\;\mathrm{FP}\;+\;\mathrm{FN}}$$

(11) $$P=\frac{\mathrm{TP}}{\mathrm{TP}+\mathrm{FP}}$$

(12) $$R=\frac{\mathrm{TP}}{\mathrm{TP}+\mathrm{FN}}$$

(13) $$F1=\frac{2\times P\times R}{P+R}$$

where TP denotes true positives, TN denotes true negatives, FP denotes false positives, and FN denotes false negatives. In the hand hazardous-zone intrusion detection task, the positive class is defined as the hazardous condition, indicating that the hand has entered the predefined danger zone.

Accuracy reflects the overall classification correctness of the proposed method. Precision indicates the proportion of correctly identified hazardous samples among all samples predicted as hazardous, whereas Recall measures the proportion of actual hazardous samples that are successfully detected. The F1-score, defined as the harmonic mean of Precision and Recall, provides a comprehensive assessment of detection performance.

## 3. Results

**Figure 16:**
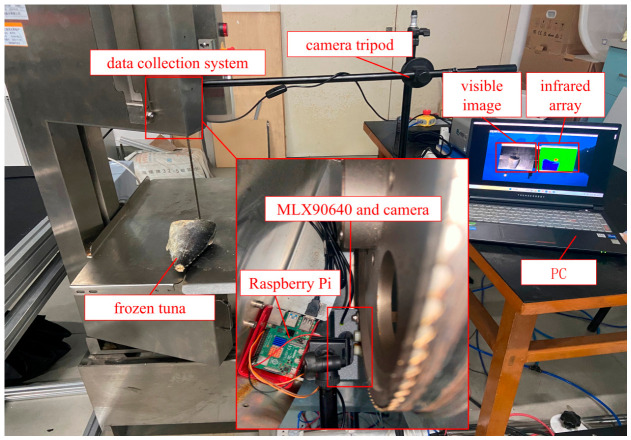
Experimental scenario.

Due to on-site production safety regulations and operational constraints of industrial equipment, it is infeasible to halt or disassemble a commercial frozen tuna cutting production line for destructive or repetitive sampling experiments. To ensure controllable data acquisition and experimental reproducibility, this study employs the Naihui JG300-NK1.5 band-saw boning machine to construct an indoor simulated experimental platform. The experimental scenario is shown in Figure 16. The thermopile sensor and the USB camera were installed above the cutting plane of the cutting machine, facing the cutting platform to capture the thermal distribution.

### 3.1. Hand Detection

Based on the 24 × 32 resolution temperature data acquired by the MLX90640 infrared thermopile sensor, experimental tests were conducted through the procedures of infrared data preprocessing, filtering and denoising, background construction, and hand detection. Notably, the infrared thermopile sensor began displaying the infrared array image only after the acquisition of eight frames of empty-scene data, ensuring that the background model was properly initialized before any target detection was performed.

#### 3.1.1. Empty Scene

**Figure 17:**
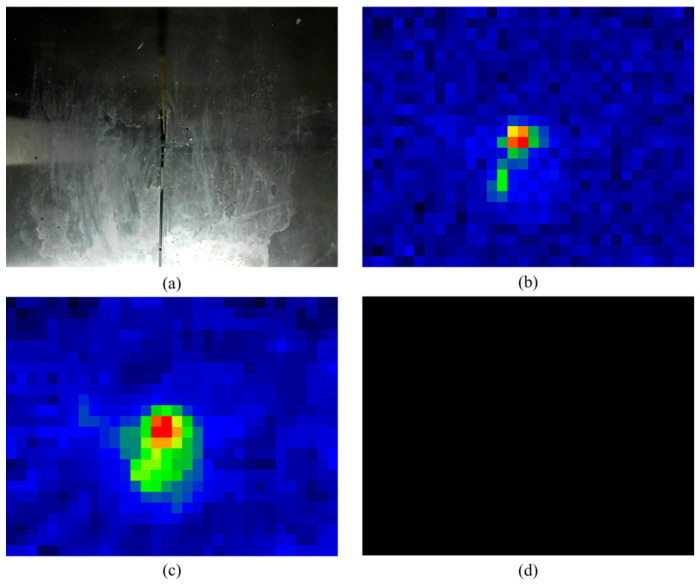
Empty scene experimental results (the heatmap represents temperature distribution, where warmer colors indicate higher temperatures and cooler colors indicate lower temperatures). (**a**) Real scene photograph; (**b**) Infrared array image after defective pixel removal only (no filtering); (**c**) Infrared array image after filtering (defective pixel removal + median filtering + EMA); (**d**) Hand detection result (no foreground detected, as expected).

In the empty scene scenario, no target appears within the detection range of the infrared thermopile sensor, and only the environmental background is present. The experimental results are shown in Figure 17. In the frozen tuna cutting scenario, the establishment of a reference background is essential. During the experiments, it was observed that there are gaps in the band-saw installation on the cutting plane, and the temperature of these gaps is higher than that of other areas on the cutting plane, approaching the temperature of the human hand. Since the positions of these high-temperature gaps are fixed, the reference background established by acquiring eight frames without any target can effectively account for these gaps, preventing them from being misidentified as foreground targets.

#### 3.1.2. Frozen Tuna Only

**Figure 18:**
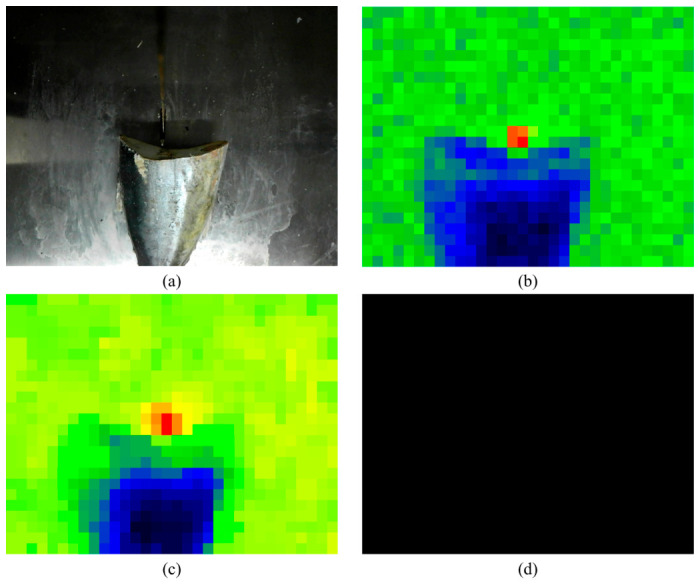
Frozen-tuna-only scenario experimental results (the heatmap represents temperature distribution, where warmer colors indicate higher temperatures and cooler colors indicate lower temperatures). (**a**) Real scene photograph; (**b**) Infrared array image after defective pixel removal only (no filtering); (**c**) Infrared array image after filtering (defective pixel removal + median filtering + EMA); (**d**) Hand detection result (no foreground detected, as expected).

In the scenario where only frozen tuna appears, only the frozen tuna and the environmental background are present within the detection range of the infrared thermopile sensor. The experimental results are shown in Figure 18. As illustrated, the frozen tuna exhibits a temperature significantly lower than the ambient environment. Due to the temperature lower-limit filtering mechanism (threshold set at 5 °C), the frozen tuna is correctly excluded from being identified as a foreground target, resulting in no false detection.

#### 3.1.3. Gloved Hand Only

**Figure 19:**
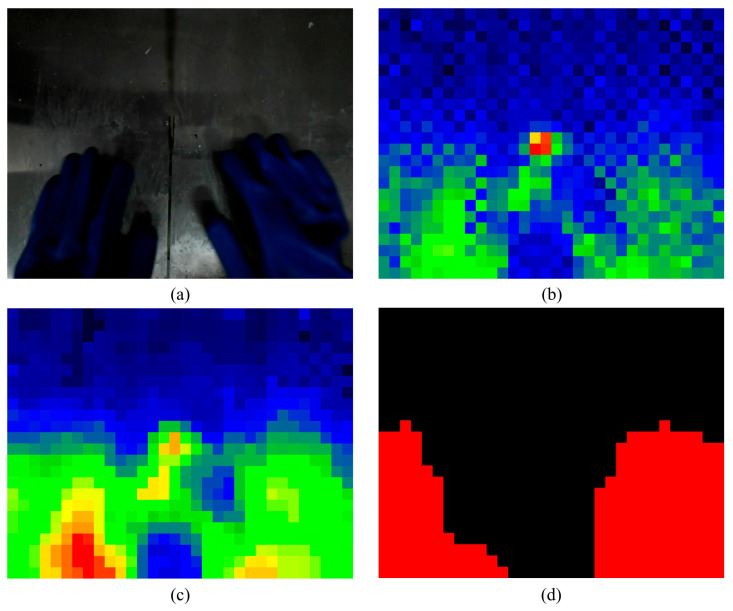
Gloved-hand-only scenario experimental results (the heatmap represents temperature distribution, where warmer colors indicate higher temperatures and cooler colors indicate lower temperatures). (**a**) Real scene photograph; (**b**) Infrared array image after defective pixel removal only (no filtering); (**c**) Infrared array image after filtering (defective pixel removal + median filtering + EMA); (**d**) Hand detection result (foreground detected, as expected).

In the scenario where only a gloved hand appears, only the gloved human hand and the environmental background are present within the detection range of the infrared thermopile sensor. The experimental results are shown in Figure 19. As illustrated, the gloved hand exhibits a temperature higher than the background reference. Despite the thermal attenuation caused by the glove material, the temperature of the hand region remains sufficiently above the background to exceed the lower-limit constraint (5 °C). Consequently, the proposed method successfully identifies the gloved hand as the foreground target.

#### 3.1.4. Gloved Hand and Frozen Tuna

**Figure 20:**
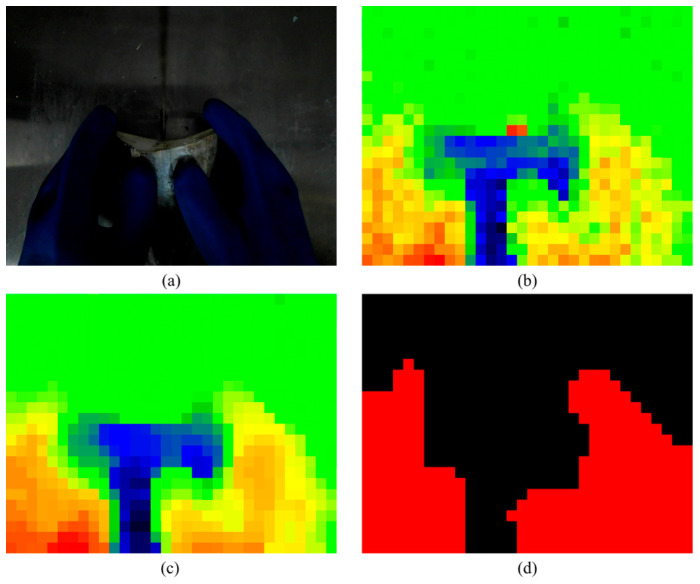
Gloved hand and frozen tuna scenario experimental results (the heatmap represents temperature distribution, where warmer colors indicate higher temperatures and cooler colors indicate lower temperatures). (**a**) Real scene photograph; (**b**) Infrared array image after defective pixel removal only (no filtering); (**c**) Infrared array image after filtering (defective pixel removal + median filtering + EMA); (**d**) Hand detection result (foreground detected, as expected).

In the scenario where a gloved hand and frozen tuna appear simultaneously, the gloved human hand, the frozen tuna, and the environmental background are all present within the detection range of the infrared thermopile sensor. The experimental results are shown in Figure 20. As illustrated, the gloved hand exhibits a temperature significantly higher than the background, while the frozen tuna shows a temperature considerably lower than the background. Due to the temperature lower-limit filtering mechanism (threshold set at 5 °C), the frozen tuna is correctly excluded from being identified as a foreground target. Meanwhile, the gloved hand, despite thermal attenuation caused by the glove material, maintains a temperature above the detection threshold and is successfully detected as the foreground target. These results validate the effectiveness of the proposed method in distinguishing between the human hand and cold objects.

To validate the effectiveness of the proposed hand detection method, the following experimental procedure was conducted. After acquiring eight frames of empty-scene data to establish the reference background, no object was initially placed within the sensor’s field of view. Under this condition, the infrared array for human detection showed a blank image. Subsequently, frozen tuna was placed within the sensor’s field of view, and the infrared array remained blank. The frozen tuna was then removed, and a gloved human hand was placed within the sensor’s field of view. At this point, the shape of the human hand appeared in the infrared array. Finally, a gloved hand holding frozen tuna was placed within the sensor’s field of view. Under this condition, the infrared array successfully displayed the hand region while not showing the frozen tuna. Although the boundary of the detected hand in the infrared array was not perfectly accurate due to the low resolution of the thermopile sensor (24 × 32 pixels), the complete hand target was still clearly displayed, demonstrating the practical feasibility of the proposed method for hand detection in frozen tuna cutting scenarios.

### 3.2. Hazardous-Zone Hand Intrusion Detection

To evaluate the performance of the proposed method for hazardous-zone hand intrusion detection in frozen tuna cutting scenarios, an infrared dataset was constructed based on a laboratory-simulated cutting platform, and corresponding intrusion detection experiments were conducted.

**Figure 21:**
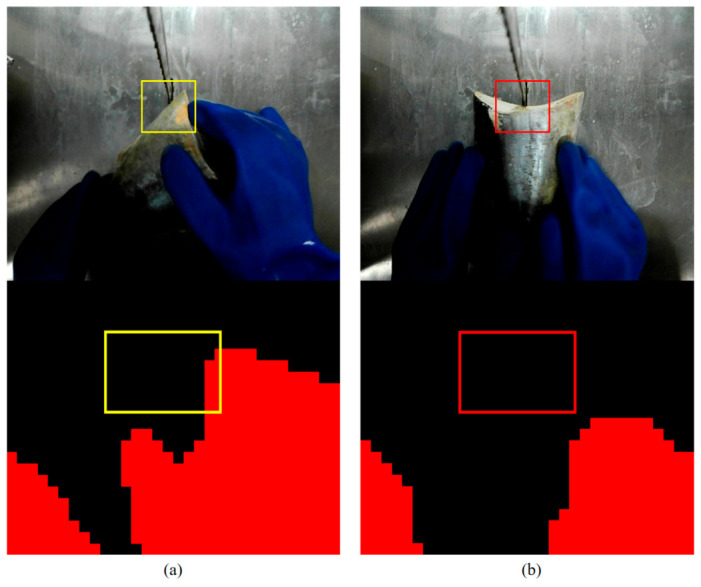
Examples of hazardous and safe samples. (**a**) Hazardous sample, the hazardous zone boundary is shown in yellow; (**b**) Safe sample, the hazardous zone boundary is shown in red.

Temperature data were acquired using the MLX90640 thermopile array sensor, with each frame represented as a 24 × 32 temperature matrix. All data were stored in text format. After data screening and annotation, a total of 1173 valid frames were obtained, comprising 611 hazardous samples and 562 safe samples. The relatively balanced distribution of positive and negative samples effectively mitigates the influence of class imbalance on experimental outcomes and enhances the objectivity of the evaluation results. Hazardous samples correspond to situations in which the operator’s hand intrudes into the predefined hazardous zone, indicating a potential safety risk. In contrast, safe samples represent normal operating conditions in which the hand remains outside the hazardous area. Figure 21 shows representative examples of both categories. As illustrated in Figure 21a, the detected hand region overlaps with the predefined hazardous zone, resulting in a hazardous intrusion event. Conversely, in Figure 21b, the hand region remains outside the hazardous zone and is therefore classified as a safe sample. These examples demonstrate the visual differences between the two operating conditions and provide a basis for evaluating the effectiveness of the proposed intrusion detection strategy.

**Figure 22:**
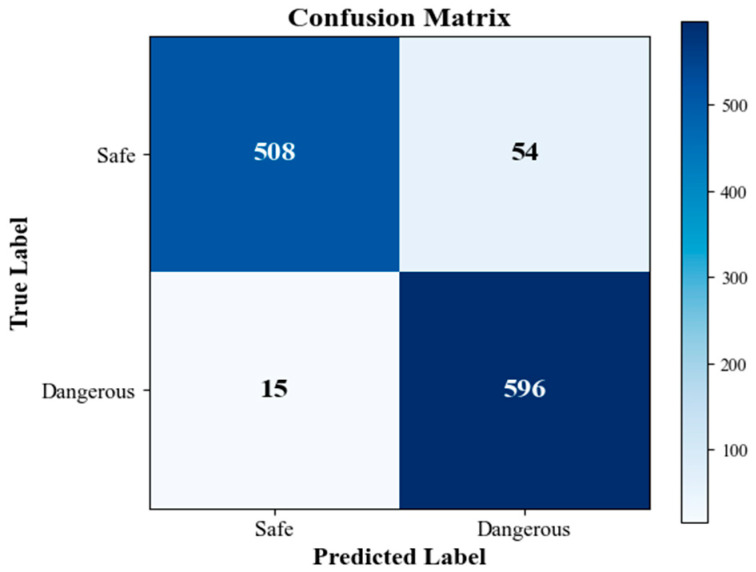
Confusion matrix of hazardous-zone hand intrusion detection.

To evaluate the effectiveness of the proposed method for hand intrusion detection in hazardous zones, the algorithm performance was assessed using the metrics defined in Section 2.9, namely Accuracy, Precision, Recall, and F1-Score. The evaluation was conducted on the entire dataset of 1173 frames. The resulting confusion matrix derived from the test is presented in Figure 22. The diagonal elements represent correctly classified samples, including hazardous intrusion events that were successfully detected and safe conditions that were correctly identified, whereas the off-diagonal elements correspond to misclassification cases, including false alarms and missed detections. The predominance of correctly classified samples along the diagonal demonstrates that the proposed method can effectively distinguish hazardous intrusion events from safe operating conditions, thereby confirming its reliability for hazardous-zone monitoring in frozen tuna cutting environments.

As shown in the confusion matrix, 508 out of 562 safe samples were correctly classified as safe, whereas 54 samples were incorrectly identified as hazardous. For the hazardous samples, 596 out of 611 were correctly detected, with only 15 samples misclassified as safe. The relatively small number of false negatives demonstrates that the proposed method is capable of reliably identifying hazardous intrusion events and maintaining a low missed-detection rate.

Based on the confusion matrix, the performance metrics defined in Section 2.9 were further calculated, and the results are summarized in Table 3.

The proposed method achieved an overall detection accuracy of 94.12% in the hazardous-zone intrusion detection task. The recall reached 97.55%, indicating that the algorithm can identify the vast majority of hazardous intrusion events, with a low missed-detection rate. High recall is particularly important for safety protection systems, as missed detections may expose operators to hazardous conditions without timely warning. The precision was 91.69%, demonstrating that false alarms are relatively rare. The F1-score reached 94.53%, indicating a good balance between precision and recall.

Taken together, these results demonstrate that the proposed hand detection method based on a thermopile array sensor can accurately identify hazardous-zone intrusion while maintaining real-time performance, validating its feasibility for active safety protection in frozen tuna cutting equipment.

## 4. Discussion

The proposed DDBS-based hand intrusion detection method demonstrated promising performance on the self-built infrared dataset. Based on 1173 samples with a relatively balanced distribution of hazardous and safe conditions, the proposed method achieved an accuracy of 94.12%, a precision of 91.69%, a recall of 97.55%, and an F1-score of 94.53%. The confusion matrix analysis further revealed a low missed-detection rate, with only 15 hazardous samples incorrectly classified as safe. Such a high recall is particularly important for industrial safety applications, as missed detections may expose operators to hazardous situations without timely warning. These results indicate that the proposed method can reliably identify hazardous hand intrusion events in frozen tuna cutting environments.

The effectiveness of the proposed method can be attributed to the complementary characteristics of the Dual-Domain Background Suppression (DDBS) strategy. The spatial-domain model suppresses static thermal interference by comparing the current frame with a reference background, while the temporal-domain model highlights regions exhibiting motion-related temperature variations between consecutive frames. By incorporating sensor absolute accuracy constraints, the proposed framework effectively reduces false foreground responses caused by measurement fluctuations and environmental disturbances. Furthermore, temperature thresholding, connected-component filtering, and hole-filling operations improve the integrity and consistency of the extracted hand regions, thereby enhancing the reliability of hazardous-zone intrusion detection.

Operating on 24 × 32 temperature matrices output from the thermopile array sensor, this framework relies entirely on lightweight calculations, including background modeling, threshold segmentation and connected-component analysis, with no demand for heavy deep learning inference. Benchmarked on a Raspberry Pi 4B embedded board, the system yields an average frame latency of 1.84 ms, fully meeting real-time execution criteria for industrial safety monitoring. Its low computational overhead and modest hardware requirements render it deployable on low-power, resource-limited embedded hardware.

Thermopile array sensors provide several practical advantages, including low cost, compact size, low power consumption, non-contact operation, and privacy preservation. These characteristics make them particularly attractive for large-scale deployment in frozen food processing equipment. By combining thermopile sensing with the proposed DDBS framework, the system effectively addresses challenges associated with thermal-insulating gloves and large low-temperature regions caused by frozen tuna.

Despite the encouraging experimental results, several limitations remain. First, the dataset size is relatively limited and was collected primarily using a laboratory-scale experimental platform. Additional validation using data acquired from real industrial production environments is necessary to further evaluate the generalization capability of the proposed method. Second, the current implementation relies on a fixed sensor field of view and manually predefined hazardous zones. In dynamic industrial environments, adaptive hazardous-zone calibration and updating strategies may be required. Third, occasional false detections and missed detections may occur in complex multi-target scenarios or during rapid hand movements. In addition, the relatively low spatial resolution of the MLX90640 sensor (24 × 32 pixels) limits the amount of spatial information available for target representation. Although the proposed system is intended for hazardous-zone intrusion detection rather than detailed hand-shape recognition, very small intrusion events or partially visible hand regions near hazardous-zone boundaries may reduce detection sensitivity under certain operating conditions.

Future work will focus on long-term validation in practical industrial production lines and the investigation of multi-sensor fusion techniques and adaptive hazardous-zone modeling strategies to further improve system robustness and environmental adaptability.

It should be emphasized that the proposed thermopile-array-based DDBS method is intended solely as a low-cost auxiliary safety-monitoring and warning solution and cannot replace certified industrial machine safety systems. Due to the limited spatial resolution, sensing capability, and response performance of the thermopile array sensor, the proposed system does not provide sufficient safety integrity for use as a standalone protective device. In particular, it must not be used as the sole safety measure in industrial machinery where direct contact between operators and cutting tools may occur, as its performance may not satisfy the response-speed and reliability requirements of such applications. Therefore, practical deployment of the proposed method should always be combined with certified safety devices, safety interlocks, emergency-stop mechanisms, and other engineering protection measures required by industrial safety standards.

Overall, the proposed thermopile-array-based DDBS method demonstrates advantages in terms of low hardware cost, low computational complexity, real-time performance, and reliable intrusion detection. The experimental results indicate that the proposed approach can serve as an effective supplementary monitoring technology for frozen food processing equipment, providing an additional layer of safety awareness while complementing, rather than replacing, certified industrial safety protection systems.

## 5. Conclusions

To address safety risks caused by hand intrusion into hazardous zones during frozen tuna cutting, this study proposes a real-time intrusion detection method based on an MLX90640 thermopile array infrared sensor. A Dual-Domain Background Suppression (DDBS) framework is developed by integrating spatial–temporal background modeling with sensor absolute accuracy constraints to robustly extract hand targets under complex low-temperature thermal environments.

Raw infrared data are preprocessed using defective pixel correction, spatial remapping, and hybrid filtering to improve signal stability. Foreground regions are subsequently extracted through joint spatial–temporal background suppression and further refined using temperature thresholding, connected-component analysis, and morphological hole filling to enhance the structural consistency of gloved hand regions. Hazardous-zone intrusion is determined by evaluating spatial overlap between detected hand regions and a pre-calibrated risk area.

The proposed method is implemented and validated on a Raspberry Pi 4B embedded platform. Experiments are conducted on a dataset of 1173 infrared frames collected from a laboratory-scale frozen tuna cutting testbed, including 611 hazardous and 562 safe samples. Results show that the proposed approach achieves an accuracy of 94.12%, a precision of 91.69%, a recall of 97.55%, and an F1-score of 94.53%, with an average processing latency of 1.84 ms per frame.

The results demonstrate that the proposed method provides a lightweight and reliable solution for real-time safety monitoring in frozen food processing environments under severe thermal contrast conditions. However, it should be emphasized that the proposed method is intended only as an auxiliary safety-monitoring and warning system and cannot replace certified industrial machine safety systems. Due to the limited spatial resolution, sensing capability, and response performance of the low-cost thermopile-array sensor, the proposed system does not provide sufficient safety integrity for exclusive use as a machine protection system. In particular, it must not be used as the sole safety measure in industrial applications where direct contact between operators and cutting tools may occur, since its performance may not satisfy the response-speed and reliability requirements of such scenarios. Therefore, the proposed method should be deployed in conjunction with certified safety devices, safety interlocks, emergency-stop mechanisms, and other engineering protection measures required by industrial safety standards.

## Figures and Tables

**Table 1:** Technical parameters of MLX90640.

Parameter	Value
Temperature measurement range	−40 °C to 300 °C
Operating temperature range	−45 °C to 85 °C
Noise equivalent temperature difference (NETD)	0.1 K (RMS @ 1 Hz)
Refresh rate (configurable)	0.5 Hz to 60 Hz
Operating voltage	3.3 V to 5 V

**Table 2:** Quantitative analysis results of multiple filtering algorithms.

Filtering Algorithm	μNRMSE (%)	σNRMSE (%)	μNRMSE Descender (%)	σNRMSE Descender (%)
No filtering	6.47	5.49	0.00	0.00
EMA	5.95	5.01	8.09	8.67
3 × 3 mean filtering	5.78	4.73	10.66	13.85
3 × 3 median filtering	3.76	3.55	41.94	35.30
EMA + mean filtering	4.06	3.85	37.33	29.79
EMA + median filtering	2.82	2.31	56.38	57.86

**Table 3:** Performance evaluation results.

Metric	Value (%)
Accuracy	94.12
Precision	91.69
Recall	97.55
F1-score	94.53

## Data Availability

The original contributions presented in this study are included in the article. Further inquiries can be directed to the corresponding authors.
